# Motivations and barriers for healthy participants to participate in herbal remedy clinical trial in Tanzania: A qualitative study based on the theory of planned behaviour

**DOI:** 10.1371/journal.pone.0271828

**Published:** 2022-07-21

**Authors:** Kamaka R. Kassimu, Florence A. Milando, Justin J. Omolo, Gloria Nyaulingo, Hussein Mbarak, Latipha Mohamed, Ramla Rashid, Saumu Ahmed, Mohammed Rashid, Gumi Abdallah, Thabit Mbaga, Fatuma Issa, Omar Lweno, Neema Balige, Bakari Mwalimu, Ali Hamad, Ally Olotu, Said Jongo, Billy Ngasala, Salim Abdulla

**Affiliations:** 1 Bagamoyo Clinical Trial Facility, Ifakara Health Institute, Bagamoyo, Tanzania; 2 Department of Parasitology, Muhimbili University of Health and Allied Sciences, Dar es salaam, Tanzania; 3 Department of traditional medicine, National Institute for Medical Research, Dar es salaam, Tanzania; Unaizah College of Pharmacy, Qassim University, SAUDI ARABIA

## Abstract

**Background:**

The success of any randomized clinical trial relies on the willingness of people to be recruited in the trial. However, 90% of all clinical trials worldwide have been reported to have failed to recruit the required number of trial participants within the scheduled time. This study aimed to qualitatively explore the motivations and barriers for healthy participants to participate in herbal remedy clinical trials in Tanzania.

**Materials and methods:**

This study used a qualitative descriptive research design based on the theory of planned behaviour. A total of five Focus Group Discussions (FGD) were conducted at Bagamoyo Clinical Trial Facility from 29 to 30 May 2021. Each group consisted of 5 to 10 participants. The participants of the study were 30 healthy males aged 18 to 45 male who participated in the clinical trial that evaluated the safety, tolerability, and efficacy of Maytenus Senegalensis. The focus group discussions were recorded audio-recorded. Verbatim transcription and thematic analysis were performed on the data.

**Results:**

The prominent motivations mentioned were the opportunity for self-development, altruism, flexible study visit schedule, and financial compensation. Furthermore, the Participants’ mothers and friends were reported as those most likely to approve of participation in an herbal remedy. The most mentioned barriers were inconvenience related to time commitment requirements, possible side effects, inflexible study visit schedule, and having other commitments. Moreover, the participants’ father was reported to be more likely to disapprove of participation in a clinical trial of herbal remedy clinical trial.

**Conclusions:**

The results of this study showed that the motivations and barriers of healthy participants to participate in clinical trials of herbal remedies are varied and that participants are motivated by more than financial gains. The identified motivations and barriers can be used as a guideline to improve the design of recruitment and retention strategies for herbal remedy clinical trials.

## Introduction

The use of Traditional herbal medicine as a treatment of various diseases and health conditions has a very long historical background [1]. In Africa, traditional herbal is much more prevalent compared to conventional medicine, and around 80% of people in African countries are regular users [2].

Herbal medicines, also called botanical medicines, vegetable medicines, or phytomedicines. As defined by World Health Organization (WHO), herbal medicines are herbal materials, herbal preparations, and finished herbal products that contain as active ingredients parts of plants, or other plant materials, or combinations as active ingredients [3]. Despite its wide use, there is a dearth of clinical evidence supporting the safety and efficacy information for most traditional herbal medicines.

Randomized clinical trials (RCTs) are seen as the gold standard for the evaluation of medical products. The introduction of randomized clinical trials in the evaluation of traditional herbal medicines could open up the possibility of testing many herbal products in Africa. The success of any randomized clinical trial depends on the willingness of people to be recruited in the trial. However, 90% of all clinical trials worldwide have been reported to have failed to recruit the required number of trial participants within scheduled time [4].

Sluggish recruitment for clinical trials adversely affects the timely accrual of clinical data. Therefore, the delay in obtaining of clinical data could lead to a further delay in the development of a medical product or result in the abandonment of a product before its true value is known [5–11].An understanding of the motivation of clinical trial participants to participate in the trials is, therefore, imperative.

Previous studies have been suggested several potential influences or motivation factors for the participant’s decision to participate in randomized clinical trials. These include the perception of study procedures, clinical trial staff, the travel times during study visits, altruism, expected health benefits, and financial consequences attached to the participation in the trial [12–24].

To our knowledge, no previous published studies have investigated motivations and barriers for the decision to participate in clinical trials of herbal remedies. In addition to that, most of those studies were also not based on a theoretical framework. The theoretical framework provides a general theoretical lens through which investigators approach study methods and analysis to make sense of social phenomena [25]. There is a need to explore the motivations and barriers of Healthy participants to participate in herbal remedy clinical trials of herbal remedies in Tanzania using the Theory of Planned Behaviour (TPB). This theory of planned behaviour has improved our understanding of the participants of the the motivations of herbal remedy clinical trials. Theory of Planned Behaviour (TPB) is a well-validated decision-making model and is widely used to improve our understanding of health-related human behaviour[26].

**Fig 1:**
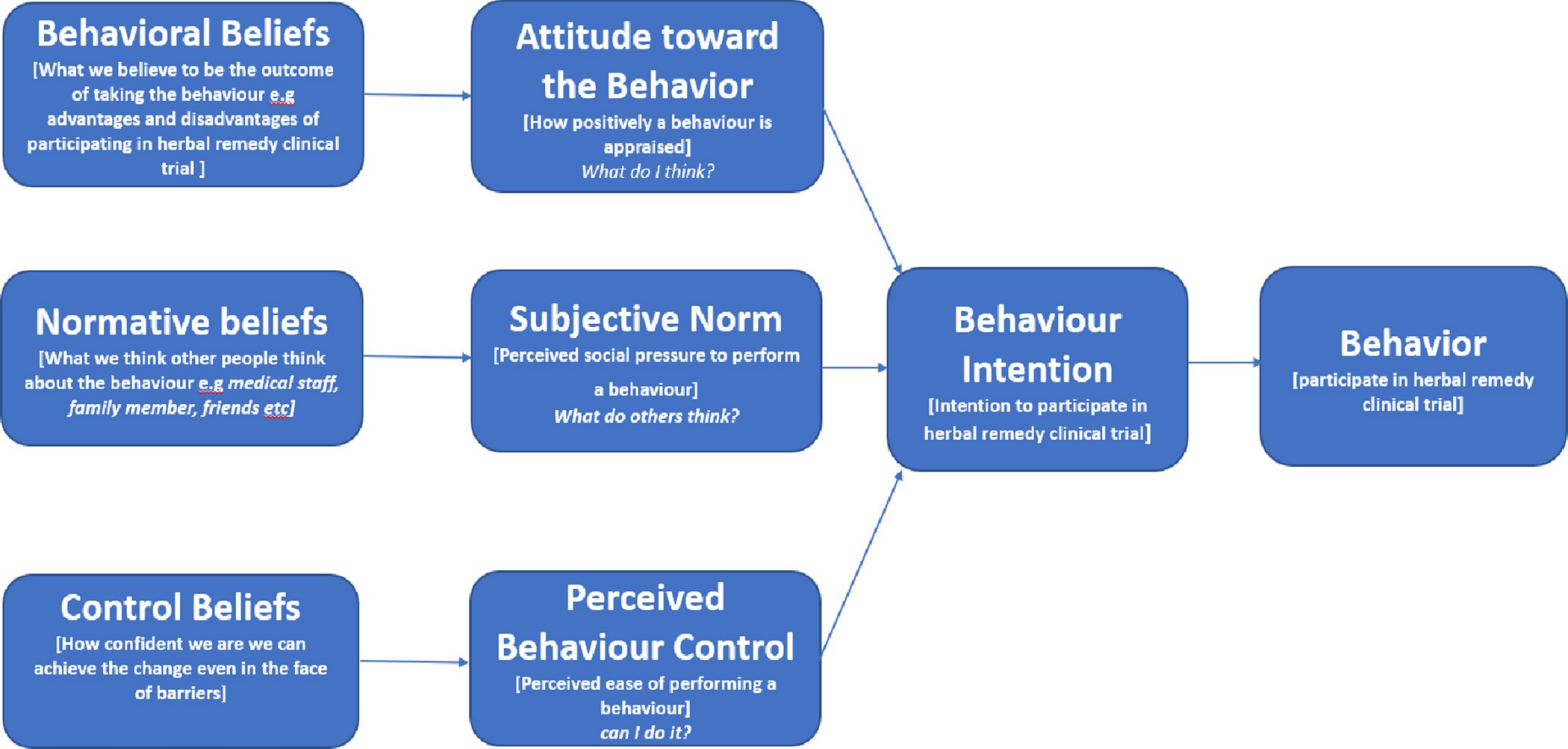
Application of the theory of planned behaviour model to investigate attitude, subjective norm, and perceived behaviour control in determine healthy participants’ participation in herbal remedy clinical trial. Adapted from Ajzen [26,27].

The Theory of Planned Behaviour (TPB) proposes that an individual behaviour is determined by intention to adopt a behaviour. Furthermore, TPB proposes three best determinants of intention to adopt a behaviour: attitude, subjective norms, and perceived behavioural control. Attitude is defined, by behavioural beliefs (i.e., perceived advantages or disadvantages of the behaviour), subjective norms by normative beliefs (i.e., social pressures to adopt the behaviour), and perceived behavioural control by control beliefs (i.e., perceived ease or difficulty of adopting the behaviour) (Fig 1) [26,27].

This study aimed to qualitatively explore the motivations and barriers for healthy participants to participate in herbal remedy clinical trials in Tanzania. In particular, the study aimed to identify the commonly held beliefs related to attitudes (behavioural beliefs), subjective norms (normative beliefs) and perceived behavioural control (control beliefs) motivations and barriers for healthy participants’ participation in clinical trials of herbal remedies.

## Materials and methods

### Study setting and population

The study was carried out at the Bagamoyo Clinical Trial Facility (BCTF), located on the Kingani Estate, 74 kilometers north of Dar-es-salaam in the Coastal region. The study population consisted of healthy male adults aged 18 to 45 years who were taking part in the first stage of a randomized clinical trial that evaluated the safety, tolerability and efficacy of *Maytenus Senegalensis*.

### Study design, sampling, and sample size

The study used a qualitative descriptive research design based on the Theory of Planned Behaviour framework to gain an in-depth understanding of the motivations and barriers for healthy participants to participate in herbal remedy clinical trials. This qualitative study was embedded within a randomized controlled trial evaluating the safety and efficacy of *Maytenus Senegalensis* (herbal remedy) to for the Treatment of Uncomplicated Malaria Episodes in Adult Patients aged 18 to 45 years old Compared to Artemether-lumefantrine (ClinicalTrials.gov Identifier: NCT04944966). Participants in the first stage of the randomized controlled trial were healthy individuals and for the second stage were patients with simple malaria.

A qualitative study within trials has been used for different purposes, including to provide insight into processes of behaviour change or to help understand the findings of the study, to identify the acceptability of the intervention or the trial protocol, and to optimize recruitment and informed consent strategies [28–31].

The sampling strategy was purposeful. All 30 participants who completed the screening procedure of the first stage of the main randomized controlled trial were invited and agreed to participate.

### Data collection

A total of five Focus Group Discussions (FGD) were conducted at Bagamoyo Clinical Trial Facility (BCTF) from 29 to 30 May 2021. Each group consisted of 5 to 10 participants (Table 2). The focus group discussions were moderated and recorded. In addition, the notes were taken by a designated note taker during discussions as a backup of the recorded data. At the end of each focus group, participants were asked if they would like to add. The duration of the focus group discussion ranged from 35 and 66 minutes.

The interview guide was developed based on relevant previous literature in the context of TPB. Interview questions were grouped into predefined sets, each related to an element of the theory of planned behaviour (TPB) (Table 1). Additionally, the interview guide was pretested for the group of people who were not part of the study participants and necessary changes were made to improve the clarity and length of the questions. The pre-tested data were not included in the results in the present manuscript.

**Table 1:** Focus group discussion guide.

TPB component	Elicited beliefs	Question
Behavioural beliefs	Advantages	“What do you think are the advantages of volunteering for an herbal remedy clinical trial?”
	Disadvantages	“What do you think are the disadvantages of volunteering for an herbal remedy clinical trial?”
Normative beliefs	Normative approval	“Consider the people important to you, who are they and would they approve of you volunteering for an herbal remedy clinical trial?”
	Normative disapproval	“Is there anyone who would disapprove of you volunteering for an herbal remedy clinical trial?”
Control beliefs	Barriers	What factors would encourage you/make it easier to volunteer for an herbal remedy clinical trial?
	Facilitators	What factors would discourage you/make it harder to volunteer for an herbal remedy clinical trial?

The interviews were conducted in Swahili language, recorded, later transcribed into Swahili transcripts. Quality check was done by listening to the audios versus transcripts. The final Swahili transcripts/results were translated into English by the researcher who conversant with both languages (Swahili and English). The quotes were back-translated to ensure the fidelity of the interviewees’ statements.

The study population for this qualitative study was limited to only 30 healthy participants who agreed to participate in the clinical trial of herbal remedies. Therefore, the data saturation point was not used to decide when to stop further focus group discussion. However, it was believed that the data saturation was reached because almost all the new themes were identified before the analysis of the fifth focus group discussion session. Furthermore, according to the previous literature, 3 to 6 focus groups are typically sufficient to achieve data saturation [32–35].

### Ethics statement

The randomized control trial in which this study was embarked, was approved by institutional review boards (IRBs) of the Ifakara Health Institute (Ref. IHI/IRB/AMM/ No: 13–2020), Senate Research and Publications Committee of Muhimbili University of Health and Allied Sciences (Ref.No.DA.282/298/01.C/30), and the National Institute for Medical Research Tanzania (NIMR/HQ/R.8a/Vol. IX/3639). The trial was also approved by the Tanzania Medicines and Medical Devices Authority (TMDA) (Ref. No. TMDA0020/CTR/0013/03). Written informed consent was sought from all participants in stage one of the randomized clinical trial (study population for this study). The signing of consent was performed after the participant had read the trial information sheet. Consent was re-affirmed verbally with participants at each Focus Group discussion and documented in the session notes by the note-taker.

### Data analysis

Data processing begins with copying the audio recordings into verbatim transcripts. The transcriptions were supplemented with notes taken during the focus group discussions. The analysis was done manually and broadly followed the six stages of thematic analysis. Thematic analysis is a method to identify, analyze and reporting patterns of meaning or theme patterns found within the qualitative data set [32]. The six steps of thematic analysis are (i) familiarisation with the data, (ii) generation of initial codes, (iii) searching for themes and patterns, (iv) reviewing themes and patterns, (v) defining and (vi) naming themes.

The theory-led approach was employed in the development of code and theme. The ordered data were reviewed and revised in discussion amongst team members and subsequently organized into themes that reflected the constructs and language of the TPB (behavioural, normative, and control belief categories). At the end of the data analysis, a content analysis was conducted on each theme and subtheme to identify the most common responses. Quotes were included in the manuscript to reflect the intended meaning of the participants.

The data analysis was conducted in the original language (Swahili) to minimize the possibility of losing the original meaning of concepts.

In this study, motivation is defined as all themes and subthemes categorized as perceived advantages, participants’ important people’s approval, and perceived ease of participation. Furthermore, the barrier is defined as all themes categorized as perceived disadvantages, participants’ important people’s disapproval, and perceived difficulty in participating in the herbal remedy clinical trial.

## Results

### Socio-demographic characteristics of participants

A total of 30 participants attended one of five focus groups ranging in size from five to 10 participants. All participants were men aged between 18 to 44 years (mean = 26.3 years). The education level of 20 out of 30 participants was secondary education. The marital status of the 22 out of 30 participants was Single. Furthermore, the occupation of 19 out of 30 participants was business. Please see Table 2 for a description of each focus group and its participants.

**Table 2:** Descriptive data of focus group participants (n = 30).

Focus group number	n	Age	Education level		Marital status		Occupation status	
**FG 1**	5	24–30 yrs.	Higher education	0	Single	2	Business	3
		Mean = 27.4	Secondary Education	5	Married	3	Peasant	0
			Primary Education	0	Co-habit	0	Fishery	0
			No education	0			Employed	2
**FG 2**	5	24–44 yrs.	Higher education	0	Single	4	Business	3
		Mean = 28.8	Secondary Education	4	Married	1	Peasant	0
			Primary Education	1	Co-habit	0	Fishery	1
			No education	0			Employed	1
**FG 3**	05	21–31 yrs.	Higher education	0	Single	4	Business	5
		Mean = 24.8	Secondary Education	3	Married	1	Peasant	0
			Primary Education	2	Co-habit	0	Fishery	0
			No education	0			Employed	0
**FG 4**	00	21–34 yrs.	Higher education	0	Single	4	Business	3
		Mean = 25.6	Secondary Education	4	Married	1	Peasant	0
			Primary Education	1	Co-habit	0	Fishery	0
			No education	0			Employed	2
**FG 5**	10	18-27yrs.	Higher education	1	Single	8	Business	5
		Mean = 25.7	Secondary Education	4	Married	2	Peasant	0
			Primary Education	4	Co-habit	0	Fishery	1
			No education	0			Employed	4
**Total**	30	18–44 yrs.	Higher education	1	Single	22	Business	19
		Mean = 26.3	Secondary Education	20	Married	8	Peasant	0
			Primary Education	9	Co-habit	0	Fishery	1
			No education	0			Employed	10

All identified themes related to health participants’ intention and behaviour to participate in herbal remedy clinical trial raised by participants in the five focus groups are categorized and displayed in Table 3 according to the three constructs of the Theory of Planned Behaviour. Furthermore, the summary of themes in Table 3 includes the number of times a theme was expressed irrespective of whether a theme was raised multiple by the same participants.

**Table 3:** Themes and subthemes matched with theory of planned behaviour components.

Behavioural Belief (Attitudes)			Normative Beliefs (Subjective norm)		Control belief (Perceived behavioural Control)	
Advantages		Disadvantages	People/groups approve	People/groups disapprove	Facilitators	Barriers
Themes	Subthemes	Themes	Themes	Themes	Themes	Themes
The opportunity for self-development	Valuable learning experience	Inconvenience related to time commitment requirements	Friends	Mother	Flexible study visit schedule	Inflexible study visit schedule and having other commitments
	Opportunity to expand social network		Spouse/partner	Sister	Financial compensation	
	Valuable life experience	Possible side effects	Other family	Brother	Novel nature of the product to be tested	Inconvenience and discomfort related to study procedure
Altruism	The desire to help others’ wellbeing		Mother	Father		
	Participate in the development of new and effective drug	Risk of not recovering after using the herbal remedy			Research staff behavior	Fear related to mistrust of clinical trial site
	Support the future of clinical trials		Father		Good track record of clinical trial site	
Participate in the development of new and effective drug						Time lost during the study visits
Access to free full medical checkup			Brother		Easy access to information about trial	Feeling unappreciated by study staff
Possibility to treat other health conditions			Uncle			
			Sister		Curiosity	Insufficient compensation
Taking part in promote the integration of herbal medicine into mainstream healthcare					Advised to participate by someone known	
Access of free to medical care						Inadequate security of the Clinical trial site
Financial reward					Low risk	

### Behavioural beliefs: Advantages and disadvantages

Participants were asked about the advantages of volunteering for an herbal remedy clinical trial. Most of them believed that the opportunity for self-development was the main advantage of participating in the herbal remedy clinical trial. Moreover, this advantage was divided into three sub-themes of (i) the valuable learning experience, (ii) the opportunity to expand the social network, and (iii) valuable life experience. Most of the participants thought that one of the advantages of participating in the herbal clinical trial was a valuable learning experience (intellectual motivation). On this, one participant said,

*“I participate for the first time*. *I have heard that my colleagues came to participate in the study and stayed for about 14 days*. *So*, *I was eager to participate in the study one day and see what it would be like to participate in the research (FGD 1*, *P2)*”

Another participant added:

“*For me*, *curiosity is what made me interested in participating in the research*. *I have many questions whenever I go to the pharmacy*. *I found a lot of drugs*, *and I wonder what these guys have mixed up*? *What have they done until they have become these drugs*? *Whenever these opportunities arise*, *I would like to come and participate because it helps me learn*. *I enjoy learning many new things (FGD 1*, *P3)”*

Furthermore, some participants thought that the opportunity to expand social networks was one of the advantages, as illustrated in the following quote.

“*Since I started participating in this study*, *it is not long*. *But I have met with doctors*. *I am already used to them*. *I have also made new friends*. *So*, *we exchange ideas with new people*. *(FGD 1*, *P2) “*

Lastly, participants thought that participation as a valuable life experience was one of advantages for participating in herbal remedy clinical trial, as seen in the following quote,

“*First of all*, *I am a history person*. *I always like to make history in certain places*. *The first thing that has led me to participate is that I want to make history*. *I know when the research is successful*, *I will be remembered for that*. *(FGD 1*, *P3)”*

Some participants mentioned that altruism is the advantage of participating in the herbal remedy clinical trial. This altruistic theme was further divided into three subthemes of (i) the desire to help others’ wellbeing, (ii) support future of clinical trials, and (iii) participation in development of new and effective drugs.

Most of participants mentioned that one of the advantages of participation is the fulfilment the desire to help others’ wellbeing, as illustrated in the following quote,

“… . *we have made a sacrifice for the community*. *After the result of this study*, *the community will benefit from us who are doing this research (FGD 4*, *P2)”*

One participant added,

“*…*… .… .… .… .*maybe in the future*. *If this drug is approved*, *it will be beneficial to my relatives and friends*…… *(FGD 5*, *P2)”*

Also, participants mentioned that support for the future of clinical trials was an advantage, as expressed by one participant,

“*If this research is completed without any problems*, *it will dispel some misconceptions in the community regarding this ongoing project*. *You will find people saying those projects that they just want you to donate blood but nothing more (FGD 5*, *P7)”*

The participants mentioned that participation in development of new and effective drug was one of the advantages, as expressed by three participants,

“*Artemether Lumefantrine causes Manyong’o nyog’o(Severe fatigue) until it is exhausted in the body*, *and then you feel better*. *But I believe that herbs will be different (FGD 3*, *P3)”*

“*The advantage of herbal medicine is that although some drugs are available and treated*. *But they do not cure some people quickly or do not cure at all*. *It means we get a natural herbal remedy that is made by tablet design without any chemicals*. *Research results may show us that it treats very effectively (FGD 1*, *P5)”*

Several participants mentioned access to free full medical check-ups as one of the advantages. One participant said,

“*The first benefit is to have a health check-up of my body and*, *I consider it to be a huge benefit*. *Health check-ups like this in other places are very expensive (FGD 2*, *P1)”*

In addition to that, one participant mentioned the possibility of herbal remedy tested in the trial might treat other health conditions as one of the advantages,

“… .… .. *herbal medicine treats malaria*, *but it can also treat other ailments like knee pain or maybe body pain (FGD 4*, *P3)”*

Some participants mentioned that taking part in promote the integration of herbal medicine into mainstream healthcare as advantage of participating in the herbal clinical, as illustrated in the following quote,

“*If it appears to be effective in this study*, *it will increase the status of herbal remedies (FGD 3*, *P4)”*

“*The second thing that interests me is that I want this research to be successful so that we can see this drug in pharmacies*. *(FGD 1, P3)*”

Some participants mentioned access free to medical care was one of the advantages. On this, one participant said,

“*In our contract*, *there is medical insurance*. *I think that’s the benefit we will get if you get sick within the project period you get free treatment (FGD 5*, *P2)”*

Only one participant mentioned financial reward as the advantage of participating in the herbal clinical trial, as seen in the following quote,

“*Everyone has their own life*. *My life as I live*, *I know myself*. *To be honest*, *I have no job to do all the time I am at Kijiweni (it is a place where unemployed people gather)*. *When I come to research*, *I earn more money than sitting at kijiweni*. *(FGD 4*, *P1)”*

When participants were asked about the disadvantages of volunteering for an herbal remedy clinical trial, most of them reported that inconvenience related to time commitment requirements as a disadvantage. One participant said,

“*For me*, *I probably see the much loss I get is time*. *Ok*, *it is a volunteer activity*, *but I see waste of time as a loss*. *Some say that time is an asset (FGD 2*, *P2)”*

Possible side effects were also mentioned as a disadvantage, and participants raising concerns about internal organ failure and other health conditions. For example, one of the participants said,

“*……*.. *you have tested me and found I have no problem*, *but after using this herb*, *you find maybe one kidney is failing to function…*… .… .… .… .… .…….*leg being swollen (FGD 4*, *P3)”*

Lastly, one participant mentioned a risk of not recovering after using the herbal remedy and getting severe malaria disease as a disadvantage,

“*…*… .… .… . *ineffectiveness of the herb in treating malaria because it is the first time being researched…*… . *A person can get malaria and use this medicine and then not recover (FGD 4*, *P4)”*

### Normative beliefs: Approval or disapproval

Participants were asked to consider the people important to them, who among those important people would approve their participation in the herbal remedy clinical trial. Several participants reported that friends, mothers, and fathers were most likely to approve them to participate in the herbal remedy clinical trial.

“*My friend is aware of this project because he has participated in one of the previous projects*. *So even as I told him*, *he said that even I would participate*, *but I have more stuff to sort out*. *So*, *you can participate*, *there is no problem*. *(FGD 1*, *P2)”*

“*My mother allowed me to participate*.… .… .… .… .… .… . *She is a good user of herbal medicine (FGD 4*, *P5)”*

“*My dad loves herbs*.…….*when he suffered from diabetes did not go to the hospital*, *he has used herbs*… .*” (FGD 4*, *P3)”*

Participants were asked to mention important people to them who would disapproval their participation in the herbal remedy clinical trial. Some of the participants reported that father, mother, sister, and brother were most likely to disapprove their participation in the herbal remedy clinical trial. Participants’ responses revealed family comes first. The family members served as important groups that may negatively affect participants decisions, as illustrated in the following quotes,

“*My father will refuse because people have built up the belief that malaria projects suck blood (FGD 3*, *P1)”*

“*I start with my mom*. *The way I see her*, *she won’t agree right away*. *She won’t say go or don’t go*. *There are some questions she will ask me*, *and I will have to respond*. *If she understands*, *she will let me participate (FGD 1*, *P4)”*

“*My sister*, *if I go to tell her that I am going to participate in the study at malaria project*. *First*, *she will say that it is up to you*. *I know there*. *But her knowledge is based on information circulated in the community*. *(FGD 1*, *P4)”*

“*My …*… .…….*brother*, *during the first research they refused to allow me to participate (FGD 1*, *P5)”*

### Control beliefs: Facilitators and barriers of behaviour

When participants were asked about factors that would encourage them or make it easier for them to volunteer for an herbal remedy clinical trial, flexible study visit schedule was most reported as a facilitator. One participant said,

“*The schedules make it easier to participate in this study because they do not command us*. *In fact*, *it considers our activities*. *(FGD 1*, *P1)”*

Financial compensation was considered a motivator to encourage participation in the trial, as illustrated in the following quotes,

“… .… .… .… ..*let me tell you the truth the thing that will keep us participating in this project is increasing the amount of compensation (FGD 5*, *P2)”*

“*We are spending too much time at the center and leave your activities unattended*. *So*, *this compensation for our time has made it easier even if you left your things*, *but you feel like you’re doing your personal activities*. *(FGD 3*, *P4)”*

The novel nature of the product to be tested (herbal remedy) also seen to facilitate participation in the trial, as expressed by one participant,

“*The thing that made me participate in the study is the news that now herbal medicine is being researched*. *This information inspired me to participate in order to find out how it helps and how it will make our life easy*? *(FGD 1*, *P5)”*

Research Staff behaviour was mentioned as a facilitator of participation in the trial. In this regard, one participant said:

“*Having collaborations with research doctors as people you have known for a long time encouraged you to participate (FGD 1*, *P2)”*

The good track record of the clinical trial site was mentioned by participants as a facilitator to the participation in the clinical trial,

“*The first thing that really motivates me to participate is that I know many people who participate in this study*. *Although I participated for the first time*, *my colleagues who participated in the previous study are ready to participate in this study*. *(FGD1*, *P4)”*

“*…the studies conducted here are going well*. *If I had heard the people participating in the research had gotten into trouble*, *I would not have come (FGD 4*, *P2)”*

Easy access to trial information made some participants feel comfortable to participate in the trial, as illustrated in the following quotes.

“*We have already read the information that anything may happen because human beings are different*. *Some people may use it without getting any effect until they finish*. *So*, *if that happens*, *I will see it as just a normal result because everyone participant in research has their own health status*. *(FGD 1*, *P2)”*

“*After being introduced to the study in the community and after coming here and reading us all the information forms*. *I find it easy to participate*. *I find that none of the features was very difficult or very harmful (FGD 2*, *P3)”*

The thing that made it easier for participation, as mentioned by participants, was curiosity. Participants wanted to see how the clinical research is conducted as said by one participant,

“*Something that made it easy to participate is that I never got participated in the research*. *So*, *when I heard the news about research and came to see for myself (FGD 3*, *P1)”*

One participant mentioned being advised to participate by someone known as the facilitator for the participation in the herbal remedy clinical trial, as illustrated in the following quote,

“*The ones that made it easier for me to participate were my friends who had participated in the research*. *They told us that participation in the research is a good thing (FGD 3*, *P3)”*

Lastly, a participant reported perception that the herbal clinical trial is a low-risk study, as a facilitator for the participation. In the words of the participant,

“*If the drugs had not been used anywhere*, *we would have used them for the first time*. *We would have been a little scared*. *But the drugs have been used in society as herbal remedies*. *Most people have used them and there are no problems*. *(FGD 2*, *P4)”*

When participants were asked about factors that would discourage them or make it harder for them to volunteer for a clinical trial of herbal remedies, the inflexible study visit schedule and having other commitments were most reported as barriers for participation in the clinical trial. As illustrated in the following quotes,

“*A rigid study schedule can make it harder to participate…*… .… .… .… .*The difficulty comes when you have an emergency the day of your study visit*. *For example*, *your parent or child is sick*. *It will not be easy to participate on that day if the schedule is unchanged (FGD 3*, *P4)”*

“*It is possible that I was scheduled to come at the same time*. *I have my responsibilities which would probably make it difficult for me (FGD 4*, *P4)”*

Inconvenience and discomfort related to study procedure also mentioned by participants as a barrier for the participation in herbal remedy clinical trial, as seen in the following quotes,

“*…*… .… .… ..*is the study procedure*. *For example*, *nurses may miss a vein during blood collection more than three times and cause unnecessary pain (FGD 4*, *P3)”*

“*The difficulty may arise due to communication problems perhaps the phone run out of air-time credit communication with the study team becomes difficult*. *(FGD1*, *P4)”*

Fear related to mistrust of the clinical trial site about the malaria study at the clinical trial site was mentioned as the barrier for participation in the herbal clinical trial. One participant said,

“*There are many bad words on the street about the study*, *people say that if you participate in the study*, *your blood will be sucked or you will be put in a room with mosquitoes*. *I did not know the truth*. *So*, *when you participate in the study*, *you find a different story (FGD 1*, *P5)”*

Some participants deterred by the time lost during the study visits, as expressed by one participant,

“*For me maybe the difficulty would be coming here*, *for example from seven o’clock in the morning until two o’clock in the afternoon because some of us have our job of carrying passengers by motorcycle*. *And these motorcycles are owned by other people (FGD 2*, *P4)”*

Feeling unappreciated by study staff was mentioned as a barrier for the participation in the herbal clinical trial,

“*The only thing that can discourage me from participating in research is lack of appreciation*… .… .… .*If you (staff) are not value participants*, *it breaks a person’s heart*. *(FGD 3*, *P5)”*

Only one participant was reported to have insufficient compensation as a barrier to participation in the clinical trial, as seen in the following quote,

“*If you stay here for two days*, *your family asked to send money…*……*I do not know if I will be able to afford my responsibilities (FGD 5*, *P8)”*

Lastly, a participant mentioned the inadequate security of the clinical trial site as an obstacle to participation in the herbal clinical trial. In the words of the participant,

“*Strict security oversight for the research center and us as a whole…*… .…….*because this facility is located in an isolated area…*… .… .… .… .… .……*Theft can occur*, *and then you come to involve the participants in the theft*. *Because throughout the period when the participants were not here*, *there was no theft that occurred and could lead to participants being involved in that theft (FGD 5*, *P7)”*

In summary, the identified motivations for healthy participants to participate in herbal remedy clinical trials were (i) the opportunity for self-development, (ii) altruism,(iii) participation in the development of the new and effective drug,(iv) access to free full medical checkup,(v) possibility of treat other health conditions,(vi) taking part in promoting the integration of herbal medicine into mainstream healthcare, (vii) access of free to medical care,(viii) financial reward, (ix) flexible study visit schedule, (x) financial compensation, (xi) novel nature of the tested product, (xii) research staff behaviour, (xiii) a good track record of clinical trial site, (xiv) easy access to information about the trial, (xv)curiosity, (xvi)advised to participate by someone known,(xvii) low risk and (xviii) Participants’ mothers and friends approval to participate.

The identified barriers for healthy participants to participate in herbal remedy clinical trials were (i) inconvenience related to time commitment requirements, (ii) possible side effects, (iii) risk of not recovering after using the herbal remedy, (iv) inflexible study visit schedule and having other commitments, (v) Inconvenience and discomfort related to study procedure, (vi) fear related to mistrust of clinical trial site, (vii) time lost during study visits, (viii) feeling unappreciated by study staff, (ix) Insufficient compensation, and (x) inadequate security of the Clinical trial site and participants’ father disapproval to participate.

## Discussion

To our knowledge, this is the first study in Tanzania to use the Theory of Planned Behaviour to qualitatively explore the motivations and barriers for healthy participants to take part in the clinical trial of malaria herbal remedies. According to the TPB, to predict an individual’s intention to perform a certain behaviour, it must be known whether the individual is in favor of performing the behaviour (behaviour beliefs), how much the person feels social pressure to do it (normative beliefs) and whether the person feels in control of the action (control beliefs) [36]. In general, the findings of this study revealed that the motivations and barriers of healthy participants to participate in clinical trials are varied.

### Behavioural beliefs

The perceived advantages expressed by the participants were positive feelings and encouragements to participate in the herbal remedy clinical trials. Similarly, the perceived disadvantages reported by the participants were the negative feelings towards participating in the herbal remedy clinical trial. Usually, when people have negative feelings and evaluations of a particular behaviour, the ultimate intention to perform the behaviour will be diminished. As intentions are considered precursors of behaviour, the diminished intentions to perform the behaviour will result in a limited adoption of that behaviour.

The majority of participants in this study perceived that opportunity for self-development was the main advantage of participating in the remedy clinical trial. This finding is very similar to other studies that have reported that the main motivators of participants of the clinical trial participants were the opportunity to learn, the opportunity to increase social contact, and perceived participation as a valuable life experience [37–39]. The desire to learn as a motivation factor can be explained by the fact that more than two-thirds of the participants in this study had completed secondary or higher education.

Several participants in this study mentioned altruism as one of the advantages of participation in the herbal remedy clinical trial. The finding of this study mirrored the findings of other earlier studies and supported previous claims that altruism is one key driver of participation in the clinical trial [40–45]. Altruism has been defined in the number of previous literatures as the desire to help others well-being, support the future of clinical trials and medical advances [40,46–52]. In a nutshell, altruism is the undertaking of good acts without the thought of personal reward. In an effort to explain the reasons for altruism as the motivation among participants, it has been stated that these participants are more likely to be optimistic, grateful and spiritual. In addition, they are more likely to be married and have somewhat lower stress and fewer or no medical conditions. They are able to consider helping others because they are less occupied with their own health problems [53].

Several participants in this study mentioned access to free full medical check-ups as one of the advantages of participating in the clinical trial. The results of this study are consistent with other studies exploring the motivation factors for healthy participants to take part in clinical trials [51,54]. Many of these participants considered the medical check-up performed as a requirement for the Phase I screening process for the early phase clinical trials a benefit because it confirms their health status.

Furthermore, other participants thought access to free medical care was one of the advantages. This study finding is not isolated. The findings of previous studies reported that access to free better quality free medical care is an important motivation for clinical trial participation [40,55–58]. In a developing country such as Tanzania, whereas the coverage of health insurance is low [59] and poor service delivery, it is justifiable for participants to mention free medical check-ups and care in the clinical trial as a benefit.

Some participants thought that taking part in the effort to promote the integration of herbal medicine into mainstream healthcare was an advantage. This finding is not surprising because traditional herbal in Africa is much more prevalent than conventional medicine, and around 80% of people in African countries are regular users [2]. The finding of the previous studies revealed that if participants considered the tested product in clinical trial represents a solution problem that holds particular relevance to their lives, this is an important motivation in favor of clinical trial participation [47,57].

Participants had not reported many disadvantages of participation in an herbal remedy clinical trial. However, the most prominent perceived disadvantage reported was inconvenience related to time requirements. The findings echo previous literature, which stated that participants’ willingness to participate in the clinical trial increased with the reduced inconvenience related to clinical trial participation [51]. However, participants in another previous study were less likely to view inconvenience related to time requirements for participation in clinical trial as an excuse for not volunteering [60]. The finding can be interpreted that although clinical trial sites pay healthy participants as compensation for the time lost during study visits, they should implement methods that could increase efficiency and reduce participant inconvenience related to time.

One participant mentioned a risk of not recovering after using the herbal remedy and getting the severe disease as a disadvantage of participating in the herbal clinical trial. The finding in this study is consistent with the study carried out among participants of herbal medicine clinical trials. The study revealed that worrying about the quality and efficacy of herbal remedies diminished participants ‘decreased the motivation to participate in the clinical trial [61]. This finding suggested that the provision of reassurance to the study participant about the presence of close follow-up of participants for the early sign of treatment failure as part of the study procedures. In addition, participants will receive rescue therapy in case of an exacerbation of symptoms or an insufficient therapeutic effect of the tested product. Furthermore, participants should be aware that the tested product may not be effective for every individual.

Possible side effects were also mentioned as a disadvantage, with participants raising concerns about the failure of internal organs and other health conditions. The finding of this study is not isolated is in line with other studies. These studies found that fear of a new drug’s potential side effects was a potential barrier to active participation in the trials [20,62,63]. This finding suggested that participants should be aware that a side effect is generally regarded as the therapeutic effect that occurs when treatment goes beyond the anticipated effect or a problem occurs in addition to the anticipated therapeutic effect. Side effects are studied during clinical trials to weigh the benefits of the drug versus the risks. Side effects may vary for each individual depending on the individual’s age, weight, gender, ethnicity, disease state, and general health. In addition, the participant will be closely followed-up for the early dictation and management of side effects related to the study product.

### Normative beliefs

Participants in this study reported a variety of people as approving and disapproving of their participation in the herbal remedy clinical trial. But most of the participants were mentioned friend, mother, and father were most likely to approve, and father was the most likely to disapprove. Despite some participants perceived their father is most likely to disapprove of their participation, the belief was not strong enough to prevent these study participants from participating in the trial. Similarly, in the previous study, participants reported that significant others advised that they should not participate, they still took part in the study [51].

The finding of this study suggests the risk of influence of social pressure on study participants to participate or not participate in herbal remedy clinical trials because most of them reported that their parents and friends play an important role in approving their decision. The study participants conform to the norms of the African society in daily decision-making. Therefore, successful recruitment strategies should involve parents and friends of the potential participants of the herbal remedy clinical trial. In addition, clinical trial sites should make an effort to involve significant others in the consent process and give them the opportunity to discuss with members of the clinical trial team.

### Control beliefs

The TPB model suggests that perceived control reflects people’s confidence and capability to perform the target behaviour[36].

Several facilitators and barriers for participation in herbal remedy clinical trials were mentioned in this study. But the most-reported facilitator was a flexible schedule. The finding of this study is similar to the finding of previous studies on the participants’ motivation factors of participants for participating in clinical trials. These studies revealed that logistic factors such as flexibility constitute the reasons for the decision over clinical trial participation [37,47,57,64]. However, another study found suitable time frame was less significant in the decision-making process of the unemployed than the employed study participants [50]. The finding of this study implies that the clinical trial site and sponsors design the study protocol, which includes an appropriate time window for the study visit without compromising the quality of the study to allow flexibility for study visits. Furthermore, during study implementation, clinical trial investigators should allow flexibility of visit attendance around scheduled time points by considering individual participants’ needs.

Several participants reported financial compensation as a motivator to encourage their participation in the trial. This finding is consistent with the general trend in the literature about the financial compensation motivation for participation in clinical trials [40,65–70]. The majority of the participants (83.3%) in this study were in the age group 18 to 30 years old. According to the previous study, participants in this age category were more likely to report financial compensation as a motivator for participation than older participants [71]. Contrary to our expectations, almost all study participants did not consider financial compensation an advantage of participation in a herbal clinical trial, but several study participants considered it as an important facilitator of their participation. This observation can be explained by the previous qualitative studies, which stated that financial incentives may not be initially reported as motivation for participation in the clinical trial because it is not perceived as socially acceptable [69,70].

The novel nature of the product to be tested (herbal remedy) was mentioned as a facilitator of the participants taking part in the trial. According to the published literature review done by Van Overbeeke et al., [72] the characteristics of the investigational product influence the value of participants’ preferences decision making in the clinical trial. Furthermore, the use of an innovative approach for the treatment of a certain disease is one of those characteristics of the investigational product. This finding implies that the investigational product which presents the innovative solution to the problem of study participants is an important motivation factor for them taking part in the clinical trial.

Easy access to trial information made some participants feel comfortable taking part in this trial. This finding is similar to the study done to investigate the motivations of healthy participants in phase I clinical trials [37]. The investigators of the Randomized clinical trial should properly provide adequate information related to the purpose, procedures, potential risks, and benefits of the Randomized clinical trial during the consenting process. This information might facilitate potential participants to make an informed decision for participation in the clinical trial.

The good behaviour of research staff was mentioned, as a facilitator of participation in the trial. Previous studies revealed that the clinical trial staff to the study participants led to a good relationship between them during the trial. A good relationship can have a huge impact on the recruitment of new participants to the study through recommendations from previous participants. Not only that, but also, this relationship can lead to participants repeating participation in future studies [51,73]. The finding of this study suggests that the good behaviour of the clinical trial staff creates a good relationship and trust is an important element of the recruitment process of clinical trial participants.

Other identified facilitators for participants to take part in this study were the good track record of the clinical trial site, altruism, access to medical check, curiosity, perceived low-risk herbal clinical trial study, and advised to participate by someone known.

The most mentioned perceived barrier to participation in the clinical trial was inflexible scheduling and having other commitments. If the trial schedule is inflexible and participants have other commitments during the proposed time slot for study appointments, the chance of that participant refusing participation is high [74]. Additional barriers reported by a few participants were discomfort related to study procedure, fear related to mistrust of clinical trial site, time lost during the study visits, feeling unappreciated by study staff, insufficient compensation, and inadequate security of the Clinical trial site.

### Limitations

This study has limitations. First, this study used a qualitative approach to explore the underline motivation of participation in the herbal remedy clinical trial among 30 healthy male participants. Also, participation in this study was restricted to those participants who had already agreed to participate in the trial. It is unknown how representative these results are of those potential participants who have not participated in the herbal remedy clinical trial. They may have quite different perspectives from those who participated in the present study. Therefore, this makes the results cannot be generalized to a wider population. However, this study results can be transferred to similar contexts. Second, the results of this study are possible to be limited by biases inherent in qualitative research, such as social desirability, self-reporting, and non-response biases.

### Conclusions

The study identified commonly held beliefs linked to behavioural, normative, and control beliefs motivations and barriers for Healthy participants to participate in herbal remedy clinical trials. Overall, the findings strongly support the suggestion that the motivations of healthy participants in herbal clinical trials are varied and that participants are motivated by more than financial gains.

The practical implication of the study findings includes the need for the clinical trial site to provide training opportunity to study participants, such as entrepreneurship skills. The clinical trial site should also develop a comfortable and stimulating environment for participants to learn from each other. In addition, in order to strengthen participants’ altruism, the trial site should convey more and accurate information, when relevant, about the potential societal benefit of the tested product so that healthy participants have a broader sense of the process of the drug development and their role in that process. The clinical trial site should be careful of inflexible study visits schedule and provide more information to healthy participants about their health status. Also, the recruitment and retention strategies should be designed to include interventions that target parents and friends of the potential participants. More research of the similar designed study is necessary in different contexts and countries. Additionally, there is a need for further research to quantify the results of this qualitative research.

## References

[1] Josephine Ozioma E-O, Antoinette Nwamaka Chinwe O. Herbal Medicines in African Traditional Medicine. Herbal Medicine. 2019. doi: 10.5772/intechopen.80348

[2] Ahlberg BM. Integrated Health Care Systems and Indigenous Medicine: Reflections from the Sub-Sahara African Region. Frontiers in Sociology. 2017;2. doi: 10.3389/fsoc.2017.00012

[3] WHO. WHO traditional medicine strategy 2002–2005. Geneva: World Health Organization. Geneva, Switzerland. 2020.

[4] Desai M. Recruitment and retention of participants in clinical studies: Critical issues and challenges. Perspectives in Clinical Research. 2020. doi: 10.4103/picr.PICR_6_20 32670827 PMC7342339

[5] Watson JM, Torgerson DJ. Increasing recruitment to randomised trials: a review of randomised controlled trials. BMC Med Res Methodol. 2006;6: 34. doi: 10.1186/1471-2288-6-34 16854229 PMC1559709

[6] Campbell M, Snowdon C, Francis D, Elbourne D, Mcdonald A, Knight R, et al. Recruitment to randomised trials: strategies for trial enrolment and participation study. The STEPS study HTA Health Technology Assessment NHS R&D HTA Programme www.hta.ac.uk Feedback. Health Technology Assessment. 2007;11. Available: http://www.hta.ac.uk. 10.3310/hta11480 17999843

[7] Treweek S, Lockhart P, Pitkethly M, Cook JA, Kjeldstrøm M, Johansen M, et al. Methods to improve recruitment to randomised controlled trials: Cochrane systematic review and meta-analysis. BMJ Open. 2013;3: e002360. doi: 10.1136/bmjopen-2012-002360 23396504 PMC3586125

[8] Burns KEA, Magyarody N, Jiang D, Wald R. Attitudes and views of the general public towards research participation. Internal Medicine Journal. 2013;43: 531–540. doi: 10.1111/j.1445-5994.2011.02433.x 21241441

[9] Galli L, Knight R, Robertson S, Hoile E, Oladapo O, Francis D, et al. Using marketing theory to inform strategies for recruitment: a recruitment optimisation model and the txt2stop experience. Trials. 2014;15: 182. doi: 10.1186/1745-6215-15-182 24886627 PMC4057570

[10] McCullagh MC, Sanon M-A, Cohen MA. Strategies to enhance participant recruitment and retention in research involving a community-based population. Appl Nurs Res. 2014/02/27. 2014;27: 249–253. doi: 10.1016/j.apnr.2014.02.007 24667018 PMC4147021

[11] Brandberg Y, Johansson H, Bergenmar M. Patients’ knowledge and perceived understanding—Associations with consenting to participate in cancer clinical trials. Contemp Clin Trials Commun. 2015;2: 6–11. doi: 10.1016/j.conctc.2015.12.001 29736441 PMC5935834

[12] Paré Toe L, Ravinetto RM, Dierickx S, Gryseels C, Tinto H, Rouamba N, et al. Could the decision of trial participation precede the informed consent process? Evidence from Burkina Faso. PLoS ONE. 2013;8. doi: 10.1371/journal.pone.0080800 24260484 PMC3829938

[13] Miller FG, Brody H. A Critique of Clinical Equipoise: Therapeutic Misconception in the Ethics of Clinical Trials. The Hastings Center Report. 2003;33. doi: 10.2307/3528434 12854452

[14] van der Zande ISE, van der Graaf R, Hooft L, van Delden JJM. Facilitators and barriers to pregnant women’s participation in research: A systematic review. Women and Birth. 2018. doi: 10.1016/j.wombi.2017.12.009 29373261

[15] Tait AR, Voepel-Lewis T, Malviya S. Participation of children in clinical research: Factors that influence a parent’s decision to consent. Anesthesiology. 2003;99. doi: 10.1097/00000542-200310000-00012 14508312

[16] Vecchi Brumatti L, Montico M, Russian S, Tognin V, Bin M, Barbone F, et al. Analysis of motivations that lead women to participate (or not) in a newborn cohort study. BMC Pediatrics. 2013;13. doi: 10.1186/1471-2431-13-53 23577644 PMC3636025

[17] Maayan-Metzger A, Kedem-Friedrich P, Kuint J. Motivations of mothers to enroll their newborn infants in general clinical research on well-infant care and development. Pediatrics. 2008;121. doi: 10.1542/peds.2007-1571 18310179

[18] Tait AR, Voepel-Lewis T, Siewert M, Malviya S. Factors that influence parents’ decisions to consent to their child’s participation in clinical anesthesia research. Anesthesia and Analgesia. 1998;86. doi: 10.1097/00000539-199801000-00010 9428850

[19] Avis NE, Smith KW, Link CL, Hortobagyi GN, Rivera E. Factors Associated With Participation in Breast Cancer Treatment Clinical Trials. Journal of Clinical Oncology. 2006;24: 1860–1867. doi: 10.1200/JCO.2005.03.8976 16622260

[20] Mills EJ, Seely D, Rachlis B, Griffith L, Wu, P, Wilson K, et al. Barriers to participation in clinical trials of cancer: a meta-analysis and systematic review of patient-reported factors. The Lancet Oncology. 2006;7: 141–148. doi: 10.1016/S1470-2045(06)70576-9 16455478

[21] Manne S, Kashy D, Albrecht T, Wong Y-N, Lederman Flamm A, Benson 3rd AB, et al. Attitudinal barriers to participation in oncology clinical trials: factor analysis and correlates of barriers. Eur J Cancer Care (Engl). 2014/01/28. 2015;24: 28–38. doi: 10.1111/ecc.12180 24467411 PMC4417937

[22] Deneuve J, Mazouni C, Arnedos M, Prenois F, Saghatchian M, André F, et al. Abstract P5-13-02: Decision making from multidisciplinary team meetings to bedside: factors predicting for physicians' and breast cancer patients' acceptance of clinical trials proposed by MTMs. Cancer Research. 2012 Dec. doi: 10.1158/0008-5472.SABCS12-P5-13-02

[23] Walsh E, Sheridan A. Factors affecting patient participation in clinical trials in Ireland: A narrative review. Contemporary Clinical Trials Communications. 2016;3: 23–31. doi: 10.1016/j.conctc.2016.01.002 29736453 PMC5935836

[24] Moorcraft SY, Marriott C, Peckitt C, Cunningham D, Chau I, Starling N, et al. Patients’ willingness to participate in clinical trials and their views on aspects of cancer research: results of a prospective patient survey. Trials. 2016;17: 17. doi: 10.1186/s13063-015-1105-3 26745891 PMC4706669

[25] Grant C, Osanloo A. Understanding, Selecting and Integrating a Theoretical Framework in Dissertation Research: Creating the Blueprint for Your “House.” Administrative Issues Journal Education Practice and Research. 2014;4. doi: 10.5929/2014.4.2.9

[26] Ajzen I. The theory of planned behavior. Organizational Behavior and Human Decision Processes. 1991;50: 179–211. 10.1016/0749-5978(91)90020-T.

[27] Ajzen Icek. Theory of planned behavior with background factors. In: https://people.umass.edu/ajzen/tpb.background.html. 5 Jan 2022.

[28] Heaven B, Murtagh M, Rapley T, May C, Graham R, Kaner E et al. Patients or research subjects? A qualitative study of participation in a randomised controlled trial of a complex intervention. Patient Education and Counseling. 2006;62. doi: 10.1016/j.pec.2005.07.013 16181766

[29] Lawton J, Jenkins N, Darbyshire J, Farmer A, Holman R, Hallowell N. Understanding the outcomes of multi-centre clinical trials: A qualitative study of health professional experiences and views. Social Science and Medicine. 2012;74. doi: 10.1016/j.socscimed.2011.11.012 22236642

[30] Donovan J, Mills N, Smith M, Brindle L, Jacoby A, Peters T, et al. Improving design and conduct of randomised trials by embedding them in qualitative research: ProtecT (prostate testing for cancer and treatment) study. British Medical Journal. 2002. 10.1136/bmj.325.7367.766 PMC1124277 12364308

[31] Craig P, Dieppe P, Macintyre S, Mitchie S, Nazareth I, Petticrew M. Developing and evaluating complex interventions: The new Medical Research Council guidance. BMJ. 2008. doi: 10.1136/bmj.a1655 18824488 PMC2769032

[32] Hennink MM, Kaiser BN, Weber MB. What Influences Saturation? Estimating Sample Sizes in Focus Group Research. Qual Health Res. 2019;29: 1483–1496. doi: 10.1177/1049732318821692 30628545 PMC6635912

[33] Coenen M, Stamm TA, Stucki G, Cieza A. Individual interviews and focus groups in patients with rheumatoid arthritis: a comparison of two qualitative methods. Quality of life research: an international journal of quality of life aspects of treatment, care and rehabilitation. 2012;21: 359–370. doi: 10.1007/s11136-011-9943-2 21706128

[34] Morgan D. Focus Groups as Qualitative Research. Thousand Oaks, California; 1997. doi: 10.4135/9781412984287

[35] Guest G, Namey E, McKenna K. How Many Focus Groups Are Enough? Building an Evidence Base for Nonprobability Sample Sizes. Field Methods. 2016;29: 3–22. doi: 10.1177/1525822X16639015

[36] Francis AJJ, Eccles MPM, Johnston M, Walker A, Grimshaw J, Foy R, et al. Constructing Questionnaires Based on The Theory Of Planned Behaviour A Manual for Health Services Researchers. Direct. 2004. 0-9540161-5-7

[37] Manton KJ, Gauld CS, White KM, Griffin PM, Elliott SL. Qualitative study investigating the underlying motivations of healthy participants in phase I clinical trials. BMJ Open. 2019;9. doi: 10.1136/bmjopen-2018-024224 30647042 PMC6340482

[38] Ferguson PR. Clinical Trials and Healthy Volunteers. Medical Law Review. 2008;16: 23–51. doi: 10.1093/medlaw/fwm020 18174206

[39] Luzurier Q, Damm C, Lion F, Daniel C, Pellerin L, Tavolacci MP. Strategy for recruitment and factors associated with motivation and satisfaction in a randomized trial with 210 healthy volunteers without financial compensation. BMC Medical Research Methodology. 2015;15. doi: 10.1186/1471-2288-15-2 25559410 PMC4293827

[40] Nappo SA, Iafrate GB, Sanchez ZM. Motives for participating in a clinical research trial: A pilot study in Brazil. BMC Public Health. 2013;13. doi: 10.1186/1471-2458-13-19 23302375 PMC3554489

[41] Dayer JA, Siegrist CA, Huttner A. Volunteer feedback and perceptions after participation in a phase I, first-in-human Ebola vaccine trial: An anonymous survey. PLoS ONE. 2017;12. doi: 10.1371/journal.pone.0173148 28273130 PMC5342214

[42] O’Keeffe S, Weitkamp K, Isaacs D, Target M, Eatough V, Midgley N. Parents’ understanding and motivation to take part in a randomized controlled trial in the field of adolescent mental health: a qualitative study. Trials. 2020;21. doi: 10.1186/s13063-020-04857-3 33228744 PMC7684724

[43] Canvin K, Jacoby A. Duty, desire or indifference? A qualitative study of patient decisions about recruitment to an epilepsy treatment trial. Trials. 2006;7. doi: 10.1186/1745-6215-7-32 17163988 PMC1770934

[44] Edwards SJL, Braunholtz DA. Can unequal be more fair? A response to Andrew Avins. Journal of Medical Ethics. 2000. doi: 10.1136/jme.26.3.179 10860209 PMC1733212

[45] Harris JM. Scientific research is a moral duty. Journal of Medical Ethics. 2005;31. doi: 10.1136/jme.2005.011973 15800367 PMC1734128

[46] Cox K., Avis M. Psychosocial aspects of participation in early anticancer drug trials: Report of a pilot study. Cancer Nursing. 1996;19. doi: 10.1097/00002820-199606000-00004 8674026

[47] Villarruel AM, Jemmott LS, Jemmott JB, Eakin BL. Recruitment and retention of latino adolescents to a research study: Lessons learned from a randomized clinical trial. Journal for Specialists in Pediatric Nursing. 2006;11. doi: 10.1111/j.1744-6155.2006.00076.x 16999746

[48] Bevan E, Chee L, McGhee S, McInnes G. Patients’ attitudes to participation in clinical trials. British Journal of Clinical Pharmacology. 1993;35. doi: 10.1111/j.1365-2125.1993.tb05687.x 8443040 PMC1381516

[49] Lowton K. Trials and tribulations: Understanding motivations for clinical research participation amongst adults with cystic fibrosis. Social Science and Medicine. 2005;61. doi: 10.1016/j.socscimed.2005.03.039 15913858

[50] Grady C, Bedarida G, Sinaii N, Gregorio MA, Emanuel EJ. Motivations, enrollment decisions, and socio-demographic characteristics of healthy volunteers in phase 1 research. Clinical Trials. 2017. doi: 10.1177/1740774517722130 28783972 PMC9896431

[51] Almeida L, Azevedo B, Nunes T, Vaz-Da-Silva M, Soares-Da-Silva P. Why healthy subjects volunteer for phase I studies and how they perceive their participation? European Journal of Clinical Pharmacology. 2007;63. doi: 10.1007/s00228-007-0368-3 17891536

[52] Chu SH, Jeong SH, Kim EJ, Park MS, Park K, Nam M, et al. The views of patients and healthy volunteers on participation in clinical trials: An exploratory survey study. Contemporary Clinical Trials. 2012;33. doi: 10.1016/j.cct.2012.02.018 22405971

[53] Soule MC, Beale EE, Suarez L, Beach SR, Mastromauro CA, Celano CM, et al. Understanding motivations to participate in an observational research study: Why do patients enroll? Social Work in Health Care. 2016;55. doi: 10.1080/00981389.2015.1114064 26933943 PMC4870048

[54] Ranjan R, Agarwal N, Kapur P, Marwah A, Parveen R. Factors influencing participation of healthy volunteers in clinical trials: Findings from a cross-sectional study in Delhi, North India. Patient Preference and Adherence. 2019;13. doi: 10.2147/PPA.S206728 31819382 PMC6890181

[55] Mfutso-Bengo J., Manda-Taylor L., Masiye F. Motivational factors for participation in biomedical research: Evidence from a qualitative study of biomedical research participation in Blantyre District, Malawi. Journal of Empirical Research on Human Research Ethics. 2015;10. doi: 10.1177/1556264614559888 25742667

[56] Doshi MS, Kulkarni SP, Ghia CJ, Gogtay NJ, Thatte UM. Evaluation of factors that motivate participants to consent for non-therapeutic trials in India. Journal of Medical Ethics. 2013;39. doi: 10.1136/medethics-2012-100755 23475804

[57] Paço A, Ferreira M, Leal J. Motivations for participating in clinical trials and health-related product testing. Journal of Medical Marketing. 2015;15: 39–51. doi: 10.1177/1745790416650602

[58] Masiye F, Kass N, Hyder A, Ndebele P, Mfutso-Bengo J. Why mothers choose to enrol their children in malaria clinical studies and the involvement of relatives in decision making: Evidence from Malawi. Malawi Medical Journal. 2008;20. doi: 10.4314/mmj.v20i2.10957 19537433 PMC2748955

[59] Embrey M, Mbwasi R, Shekalaghe E, Liana J, Kimatta S, Ignace G, et al. National Health Insurance Fund’s relationship to retail drug outlets: a Tanzania case study. Journal of Pharmaceutical Policy and Practice. 2021;14. doi: 10.1186/s40545-021-00303-0 33593420 PMC7888141

[60] Greenslade JH, White KM. Beliefs Underlying Above Average Participation in Volunteerism. Australian Journal on Volunteering. 10.3200/SOCP.145.2.155-172 15816345

[61] Zheng W, Chang B, Chen J. Improving participant adherence in clinical research of traditional Chinese medicine. Evidence-based Complementary and Alternative Medicine. 2014. doi: 10.1155/2014/376058 24527045 PMC3912632

[62] Henrard S, Speybroeck N, Hermans C. Participation of people with hemophilia in clinical trials of new treatments: An investigation of patients’ motivations and existing barriers. Blood Transfusion. 2015;13. doi: 10.2450/2014.0152-14 25369591 PMC4385080

[63] Ross S., Grant A., Counsell C., Gillespie W., Russell I., Prescott R. Barriers to participation in randomised controlled trials: A systematic review. Journal of Clinical Epidemiology. 1999;52. doi: 10.1016/s0895-4356(99)00141-9 10580777

[64] Ellis PM. Attitudes towards and participation in randomised clinical trials in oncology: A review of the literature. Annals of Oncology. 2000. doi: 10.1023/a:1008342222205 11038029

[65] Mtunthama N, Malamba R, French N, Molyneux ME, Zijlstra EE, Gordon SB. Malawians permit research bronchoscopy due to perceived need for healthcare. Journal of Medical Ethics. 2008;34. doi: 10.1136/jme.2007.020461 18375686

[66] Vrhovac R, Francetic I, Rotim K. Drug trials on healthy volunteers in Yugoslavia. International Journal of Clinical Pharmacology Therapy and Toxicology. 1990;28. 2228323

[67] Edwards P, Cooper R, Roberts I, Frost C. Meta-analysis of randomised trials of monetary incentives and response to mailed questionnaires. Journal of Epidemiology and Community Health. 2005;59. doi: 10.1136/jech.2005.034397 16234429 PMC1732953

[68] Monahan T, Fisher JA. ‘I’m still a hustler’: entrepreneurial responses to precarity by participants in phase I clinical trials. Economy and Society. 2015;44. doi: 10.1080/03085147.2015.1113703 27524854 PMC4978131

[69] Fisher J.A. Feeding and Bleeding: The Institutional Banalization of Risk to Healthy Volunteers in Phase I Pharmaceutical Clinical Trials. Science Technology and Human Values. 2015;40. doi: 10.1177/0162243914554838 25914430 PMC4405793

[70] Stunkel L, Grady C. More than the money: A review of the literature examining healthy volunteer motivations. Contemporary Clinical Trials. 2011. doi: 10.1016/j.cct.2010.12.003 21146635 PMC4943215

[71] van Gelderen CEM, Savelkoul TJF, van Dokkum W, Meulenbelt J. Motives and perception of healthy volunteers who participate in experiments. European Journal of Clinical Pharmacology. 1993;45. doi: 10.1007/BF00315344 8405024

[72] van Overbeeke E, Whichello C, Janssens R, Veldwijk J, Cleemput I, Simoens S, et al. Factors and situations influencing the value of patient preference studies along the medical product lifecycle: a literature review. Drug Discovery Today. Elsevier Ltd; 2019. pp. 57–68. doi: 10.1016/j.drudis.2018.09.015 30266656

[73] Kass NE, Myers R, Fuchs EJ, Carson KA, Flexner C. Balancing justice and autonomy in clinical research with healthy volunteers. Clinical Pharmacology and Therapeutics. 2007;82. doi: 10.1038/sj.clpt.6100192 17410122

[74] Newington L., Metcalfe A. Factors influencing recruitment to research: Qualitative study of the experiences and perceptions of research teams. BMC Medical Research Methodology. 2014;14. doi: 10.1186/1471-2288-14-10 24456229 PMC3903025

